# Supplementary material for: NeuroRehabilitation OnLine: Description of a regional multidisciplinary group telerehabilitation innovation for stroke and neurological conditions using the Template for Intervention Description and Replication checklist
Source: Digit Health. 2024 May 28;10:20552076241252263. doi: 10.1177/20552076241252263 (PMC11138190; doi:10.1177/20552076241252263)
Supplement: sj-docx-2-dhj-10.1177_20552076241252263 - Supplemental material for NeuroRehabilitation OnLine: Description of a regional multidisciplinary group telerehabilitation innovation for stroke and neurological conditions using the Template for Intervention Description and Replication checklist [file sj-docx-2-dhj-10.1177_20552076241252263.docx]

NROL GRIPP2 short form

Checklist from: [GRIPP2 reporting checklists: tools to improve reporting of patient and public involvement in research - PubMed (nih.gov)](https://pubmed.ncbi.nlm.nih.gov/28768629/) This checklist includes the Patient and Public Involvement (PPI) from NROL development, implementation, and evaluation.

| **Section & topic** | **Item** |  |
| --- | --- | --- |
| 1: Aim | Report the aim of PPI in  the study | PPI were key stakeholders in the complex intervention development. The aim of PPI involvement was to provide vital insight to improve NROL quality and relevance. |
| 2: Methods | Provide a clear description of the methods used for PPI in the study | PPI members were part of the learning collaborative, with patients, carers and staff feedback shaping the intervention described.  *Summary activities:*   - Project oversight - Assist with:   - ensuring materials are accessible/aphasia-friendly/content-appropriate   - improving NROL content/delivery   - improving inclusivity e.g., alternative language resources   - developing patient volunteer roles   - developing dissemination plans   - highlighting findings most relevant to the public   - research outputs as co-authors/advisors - Support further funding applications and direction of work - Patient volunteer/s contribute to NROL delivery   NROL patients have contributed to feedback sessions and completed patient satisfaction surveys, expressing opinions about NROL delivery (e.g., technology, value,  likes/improvements) and offering thoughts on NROL’s future. To further understand patient perspective, 13 patients were interviewed, and 2 focus groups were held (n=9, 1hr each).  Regional patient and carer assurance groups involve people with lived experience of neurological conditions or carers, working at a regional level improving the quality and  accessibility of local services. NROL leads met twice with the Lancashire and South Cumbria  regional patient and carer group. Conversations focussed on how patients and carers may contribute substantially to guide subsequent NROL delivery, how more patient volunteers may |

|  |  | become involved and how their coordination and support may be situated at a regional level going forward. |
| --- | --- | --- |
| 3: Study results | Outcomes—Report the results of PPI in the study, including both positive and negative outcomes | Patient and carer involvement has influenced modifications to NROL delivered to date (e.g., addition of ‘Fatigue’ and ‘Cognitive Strategy’ groups), and refinement of NROL resources (e.g., NROL patient tech guide). Positive opinion has contributed to the conclusion that NROL is an appropriate and acceptable way to complement therapy. Furthermore, 99% of patients who have completed the multi-trust regional NROL would recommend NROL. Patients have emphasised a desire for wider implementation, with one patient saying “Invite more people. Roll it out across the country. Spread the word”. It is recognised that telerehabilitation is not for everyone, and we have endeavoured to document reasons for withdrawal to help understand these factors. Having technology assistance for patients to get online was key.  Some patients explained that they had been willing to ‘*give up*’ on the online option but that ‘*having someone on hand to support’* enabled them to *‘get online and stay online*’. |
| 4: Discussion and conclusions | Outcomes—Comment on the extent to which PPI influenced the study overall. Describe positive and negative effects | A strength of NROL is involvement of PPI in co-production, providing crucial insight to help understand NROL’s potential value. PPI particularly had a powerful role in establishing the value proposition of NROL from the patient perspective to assist continued funding and commissioning intent. Patients provided quotes and videos to complement patient outcome  data, and contributed to dissemination efforts in the form of press releases, media interviews, business reports and peer-reviewed articles etc. A challenge has been securing funding to support PPI activities, the processes and administration required to engage formally with  patient volunteers. |
| 5:  Reflections/critical perspective | Comment critically on the study, reflecting on the things that went well and those that did not, so others can learn from this experience | Things that went well:   - Co-production of the structure and content of NROL - Working relationship and power dynamics between PPI and clinicians Things that could go better: - Time and effort supporting PPI not having formal funding and recognition - Lack of diversity in PPI and limited resource for support e.g., for non-English language speakers |
